# Type I Diabetic Patients’ Perceptions of the Relationship Between Diabetes Mellitus and Periodontal Disease

**DOI:** 10.3390/healthcare13101150

**Published:** 2025-05-15

**Authors:** Marta Relvas, Flávia Gomes, Filomena Salazar, Cristina Cabral, Maria Arminda Santos, Rosana Costa, Maria Gonçalves

**Affiliations:** 1 Department of Medicine and Oral Surgery, University Institute of Health Sciences (IUCS-CESPU), 4585-116 Gandra, Portugal; marta.relvas@iucs.cespu.pt (M.R.); a28376@alunos.cespu.pt (F.G.); filomena.salazar@cespu.pt (F.S.); cristina.cabral@iucs.cespu.pt (C.C.); rosana.costa@iucs.cespu.pt (R.C.); 2 Oral Pathology and Rehabilitation Research Unit (UNIPRO), University Institute of Health Sciences (IUCS-CESPU), 4585-116 Gandra, Portugal; maria.santos@iucs.cespu.pt; 3 Associate Laboratory i4HB—Institute for Health and Bioeconomy, University Institute of Health Sciences (IUCS—CESPU), 4585-116 Gandra, Portugal; 4 UCIBIO—Applied Molecular Biosciences Unit, Translational Toxicology Research Laboratory, University Institute of Health Sciences (1H-TOXRUN, IUCS—CESPU), 4585-116 Gandra, Portugal

**Keywords:** diabetes mellitus, periodontal diseases, patient awareness, relationship

## Abstract

**Background/Objectives**: Knowledge about the relationship between diabetes and periodontitis is lacking in individuals diagnosed with type I diabetes. To enhance oral health and reduce the risk of oral diseases, healthcare professionals, both dentists and endocrinologists, should educate and motivate patients to treat periodontitis and its complications as soon as possible. The aim of this study was to assess the knowledge of type I diabetic patients about the relationship between periodontal disease and diabetes. **Methods:** A cross-sectional, single-center study was carried out on 80 patients from the Tâmega and Sousa Hospital Center, who were administered a questionnaire before they underwent an oral clinical examination. The data were analyzed using the chi-square test and independent *t*-test. **Results:** All participants were aware of at least one of the problems associated with diabetes. Furthermore, 18.8% of the patients reported not receiving any advice from their dentist, and 31.4% stated that they had been informed of its importance. Regarding the diagnosis of diabetes, 87.5% of participants felt that dentists should be involved. Advanced age, lower education levels, and alcohol consumption were associated with a higher prevalence of periodontal disease (35.6%) (*p* < 0.05). Health practitioners should advise early periodontal disease diagnosis, treatment, and oral health education, particularly for people with type I diabetes. **Conclusions:** Factors, such as advanced age and lower education levels, were associated with a higher prevalence of periodontal disease. Although many patients recognize the relationship between diabetes and dental problems, most do not discuss this with their dentist.

## 1. Introduction

Diabetes mellitus is a chronic condition caused by insufficient production or inadequate use of insulin. Insulin is a hormone needed to transport glucose from the blood to the cells, where it is used as energy [1,2].

The pathophysiology of type I diabetes mellitus involves an autoimmune reaction against the insulin-producing β-cells of the pancreas. The exact cause of this process is still not fully understood. In this condition, the body mistakenly attacks the cells in the pancreas responsible for producing insulin, leading to their destruction [1,2].

Type II diabetes mellitus is linked to physical inactivity, poor diet and obesity, and can be prevented with behavioral interventions focused on risk factors. It results from insulin resistance, which affects the proper utilisation of the insulin produced by the body in the target cells [1,2].

The World Health Organization estimates that 422 million people worldwide had diabetes in 2016, and by 2030, the condition is projected rank as the sixth most common cause of death worldwide [3,4].

In 2021, there were approximately 680 new cases of diabetes per 100,000 inhabitants in Portugal. The estimated prevalence of diabetes in the Portuguese population aged between 20 and 79 was 14.1%. In other words, around 1.1 million Portuguese in this age group are affected by diabetes [5].

Periodontal disease is a chronic multifactorial inflammatory condition caused by the accumulation of dental plaque, which affects the supporting tissues of the teeth (periodontium). It is characterized by the progressive destruction of the periodontal ligament and alveolar bone, which are responsible for holding the teeth implanted in the jaws, resulting in the loss of periodontal attachment [6,7,8].

Periodontitis affects the periodontal tissue, particularly the gums and the alveolar bone to which the teeth are attached. In advanced cases, periodontitis leads to gingival inflammation, loss of clinical attachment, radiographic evidence of alveolar bone loss, sites with deep probing depths, mobility, pathological migration, tooth loss, and reduced quality of life [6,8].

In a study carried out by Costa et al. in Portugal, out of a total of 70 type I diabetic patients, gingivitis reached a prevalence of 37.1% and periodontitis reached a prevalence of 55.7% [9].

Periodontal disease susceptibility depends on the interaction of risk factors, such as genetic predisposition, smoking, stress, and certain systemic diseases, including diabetes mellitus [1].

Studies on smokers have shown abnormalities in the regulation of proteolytic production, altered inflammatory cytokine profiles, and changes in gingival crevicular fluid in relation to immune cell function. Smoking affects the microbiota (the composition of the subgingival biofilm changes with the increased prevalence of periodontal pathogens), the immune system (delayed neutrophil recruitment and migration), and the healing capacity of the periodontium (greater collagenolytic activity is associated with fewer gingival blood vessels) [8,10].

Patients with uncontrolled diabetes are more likely to develop periodontitis compared to those with healthy or controlled diabetes [7,8].

Diabetes mellitus and periodontal disease are two chronic inflammatory conditions of a destructive and progressive nature. They are considered public health concerns due to their increasing prevalence on a global scale [11,12].

Several studies have revealed that periodontitis is more severe in diabetic adults compared to non-diabetics [6,9,13,14,15]. Research shows a two-way relationship between these two diseases. Considering this association, mutual collaboration between physicians and dentists is key to providing successful prevention and integrated treatment for both diseases [6,14,15].

Long-term hyperglycemia can result in oral symptoms, such as xerostomia, caries, periodontal disease, and a burning feeling in the oral mucosa, which may lead to early tooth loss [16].

Inadequate glycemic control is linked to loss of attachment and the destruction of periodontal tissue. Patients with type I diabetes have greater tooth loss due to the long duration of the disease. Poorer periodontal health leads to poorer glycemic control [2,15].

There is a lack of knowledge among people about the relationship between diabetes and periodontitis. To improve oral health and reduce the risk of oral diseases, healthcare professionals, both dentists and endocrinologists, should educate and motivate patients to treat periodontitis and its complications early [11,15,17].

Several studies have shown a reciprocal relationship between type II diabetes mellitus (T2DM) and periodontal disease; however, comparatively fewer studies have focused on the relationship between T1DM and periodontal health [9].

The aim of this study was to assess the knowledge of type I diabetic patients about the relationship between periodontal disease and diabetes, as well as to assess the prevalence of periodontal diseases and the risk factors in association with them.

## 2. Materials and Methods

### 2.1. Study Design

This cross-sectional, single-center study was conducted in accordance with STROBE guidelines [18]. The interventions were carried out in accordance with the Helsinki Declaration and approved by the Hospitalar Center of Tâmega e Sousa, Penafiel’s Ethical Committee (reference: 22/2022).

The sample included patients of booth genders, aged between 19 and 72 years, diagnosed with type I diabetes and attending the Endocrinology Unit of the Hospitalar Center of Tâmega and Sousa. Participants were selected through a non-probabilistic convenience sampling method. Out of 106 invited patients who met the inclusion criteria, 80 agreed to participate in the study.

Eligible participants provided informed consent and agreed to participate in the study. Exclusion criteria were individuals without a confirmed diagnosis of type I diabetes or not followed by the Endocrinology Unit, pregnant patients, and individuals undergoing oncological treatment.

### 2.2. Data Collection

Patients were invited to complete a questionnaire designed by an endocrinologist and a periodontologist, following an exhaustive review of the relevant literature [2,3,4]. The feasibility and clarity of the questionnaire were evaluated through a pre-test conducted on a convenience sample of 20 type I diabetics aged between 18 and 64 years old.

The questionnaire included 20 questions grouped into the following 5 categories: (i) demographic characteristics (age, gender, educational qualifications, professional occupation, type of health system attended); (ii) habits (smoking, alcohol, oral hygiene) (iii) general and oral health practices (perception of oral condition, annual visits to the diabetes care provider, frequency of visits to the Oral Hygienist and/or Dentist and reasons); (iv) knowledge about the association between diabetes and other diseases (receiving counselling from dental and diabetes care providers, knowledge about specific oral and systemic complications of diabetes); and (v) current and past experience of oral complications of diabetes (xerostomia and periodontal disease).

For patients who agreed to take part, the questions were asked verbally by the researcher, who noted down the answers.

### 2.3. Periodontal Examination

The study sample underwent an intraoral clinical examination, in which a periodontal chart was filled out. The variables collected using the periodontogram were as follows: periodontal probing depth (PPD), measured as the distance from the free gingival margin to the bottom of the pocket; gingival recession (REC), defined as the distance from the cementoenamel junction (CEJ) to the free gingival margin; clinical attachment loss (CAL); plaque index (PI); and bleeding on probing (BOP).

The O’Leary (1972) [19] index was used to assess bacterial plaque. This method allows us to determine the percentage of bacterial plaque using a plaque-revealing agent at six sites per tooth and aims to assess the effectiveness of oral hygiene of dental surfaces, as well as patient motivation.

The Ainamo and Bay (1975) [20] index was used to assess the percentage of bleeding on probing at six sites per tooth of each subject.

These data were recorded at six sites per tooth (mesio-vestibular, vestibular, disto-vestibular, mesio-lingual/palatine, lingual/palatine, and disto-lingual/palatine). A periodontal probe (PCP-UNC 15, Hu-Friedy, Chicago, IL, USA) was used to measure the periodontal pockets and plaque developer, a dye, was used to highlight the bacterial biofilm.

The diagnosis of periodontal disease was made according to the current classification of periodontal diseases by the American Association of Periodontology (AAP) and the European Federation of Periodontology (EFP) [8]. Gingivitis was defined when the total percentage of bleeding on probing was equal to or greater than 10% and the probing depth was less than or equal to 3 mm. Periodontitis was diagnosed when there was clinical attachment loss (CAL) greater than or equal to 2 mm in two or more non-adjacent interproximal spaces, or when the interproximal CAL was 3 mm or more on the buccal or lingual/palatal surfaces of at least 2 teeth and when marginal radiographic bone loss was evident on the peri-apical X-ray.

The periodontal examination was carried out by two periodontologists (MR/RC). To ensure repeatability between examiners, 20% of the sample had their periodontal parameters remeasured, and the results were compared with those obtained by a gold standard examiner. When assessing PPD and gingival recession (REC), the examiners’ k coefficients (within 1 mm) ranged from 0.79 to 0.93 and from 0.81 to 0.89, respectively. For repeated measurements, the intra-examiner agreement rates for PPD and REC were 0.89 to 0.95 and 0.82 to 0.91, respectively.

### 2.4. Statistical Analysis

The data were analyzed using IBM^®^ SPSS^®^ Statistics software (Statistical Program for Social Sciences), version 29.0 for Windows. Descriptive statistics were expressed as means and standard deviations for quantitative variables and as frequencies and percentages for qualitative variables. Categorical grouping variables included periodontal condition (with and without periodontitis) and age (≤25 years, ≥26 and ≤45 years, and ≥46 years). The normality of the data was initially assessed using the Shapiro–Wilk test, with no evidence of rejection of the null hypotheses. To strengthen this evaluation, we also examined skewness and kurtosis values for all quantitative variables. In both the periodontitis and non-periodontitis groups, all variables (PI, BOP, PPD, and CAL) presented skewness and kurtosis within acceptable limits (±1), indicating approximately normal distributions.

The normality of the data led us to adopt the independent *t*-test to compare periodontal indices (PI, BOP, PPD, and CAL) between individuals with periodontitis and those without. To measure the magnitude of the effect, Cohen’s d proved to be the most appropriate measure, and in this sense, the following guidelines were respected: |d| ≤ 0.20 was seen as a small effect, |d| = 0.50 was seen as a moderate effect, and |d| ≥ 0.80 was seen as a large effect. The chi-square test/Fisher’s exact test was used to assess the association between periodontal condition and variables, such as gender, age groups, smoking habits, alcohol consumption, oral hygiene habits, questions related to the role of the dentist in diabetes, and knowledge about oral health and its importance in diabetes. The significance level was set at 0.05.

## 3. Results

### 3.1. Sociodemographic Characteristics and Smoking and Alcohol Habits

Participants ranged in age from 19 to 72 years (mean = 35.9, SD = 13.8), with the majority (36.3%) aged between 26 and 45 years. Most participants were male (63.7%) and had completed 12 years of schooling, equivalent to secondary education (33.8%). A substantial proportion (75.0%) were employed as workers (Table 1).

Regarding alcohol consumption, 57.5% of participants reported drinking alcohol, with the majority (60.9%) drinking occasionally. Smoking habits showed that 21.3% of participants were active smokers; of these, 64.7% smoked up to 10 cigarettes per day, while 35.3% smoked more than 10 cigarettes per day (Table 1).

### 3.2. Oral Hygiene Habits and Perception of Oral Condition, Number of Dental Appointments, and Reasons for Not Going to the Dentist

Table 2 summarizes the oral hygiene habits and perception of oral condition, number of dental appointments, and reasons for not going to the dentist of the participants. Among the 80 participants, 93.8% reported brushing their teeth daily, with 52.5% brushing twice a day. Nearly all participants (93.8%) used both a toothbrush and toothpaste. However, the use of dental floss or interdental brushes was less common, with 72.5% of participants reporting that they did not use these tools. Among the 22 participants (27.5%) who used dental floss or interdental brushes, 10.0% did so daily, 12.5% occasionally, and 5.0% rarely.

Interdental cleaning practices were generally infrequent. The majority (77.5%) reported performing interdental cleaning rarely, while 12.5% did so occasionally and 10.0% did so daily. Tongue brushing was reported by 51.2% of participants as an always-practiced habit, while 20.0% brushed their tongue occasionally, and 21.8% did not brush their tongue at all. Additionally, 12 participants (15.0%) reported wearing dentures.

Participants were asked to rate the condition of their oral health. Of the total, 27 individuals (33.8%) considered their oral condition to be poor or very poor, while 51.2% said it was good, 11.3% rated it as very good, and 3% rated it as excellent. Regarding dental visits, 20% of participants reported visiting the dentist or hygienist only when experiencing pain, 32.5% visited once a year, 23.8% visited twice a year, and 23.8% visited more than twice a year. When asked about reasons for avoiding dental visits, the most commonly cited reason was not perceiving a need to go (45%), followed by cost concerns (25%) and unspecified reasons (17.5%). With regard to healthcare system usage, 73.8% of participants used private clinics, with 8.8% of these individuals reporting having health insurance. Meanwhile, 22.5% relied on the national health system, and 3.8% used a mixed system.

When we compared PI with tooth brushing frequency, we found that this parameter reached higher values in individuals who brushed their teeth only once a day (median = 44.65) compared to those who brushed twice a day (median = 41.11) or three or more times a day (median = 41.64), although these differences were not statistically significant.

### 3.3. Knowledge of Diabetes-Related Problems and the Importance of the Dentist

Table 3 summarizes participants’ responses regarding their awareness of diabetes-related problems and the role of dentists in managing this condition. All participants reported being aware of at least one diabetes-related issue, with twenty individuals identifying only one problem and the remainder recognizing four or more. Notably, 51.2% of participants identified a combination of symptoms, including dry mouth, bad breath, tooth decay, taste changes, bleeding gums, and delayed healing, while 13.8% were aware of only dry mouth, and 7.5% recognized all listed problems. Participants were also asked “*whether they had received advice from a health professional to adopt specific oral health precautions, such as brushing and flossing their teeth, checking their gums, visiting a dentist regularly, or consulting an oral hygienist*”. Of the respondents, 18.8% (*n* = 15) reported not receiving any advice, while 31.4% (*n* = 25) had been informed about the importance of brushing, flossing, gum checks, and regular dental and hygienist visits. Among the remaining participants (*n* = 40), 2.5% reported being advised solely on flossing, 2.5% on brushing, 13.8% on brushing and regular dental visits, and 18.8% on brushing, regular dental visits, and gum checks. To the question “*Do you talk to your dentist about diabetes?*”, the majority of participants (48.8%) said they did not, 27.5% did it sometimes and 23.8% did it often, with 87.5% considering it important for the dentist to talk about oral health and to be involved in diagnosing diabetes and 80% considering it important for the dentist to propose tests to be paid for by the patient.

### 3.4. Periodontal Condition

Regarding the diagnosis of periodontal disease, as can be seen in Table 4, 56.3% of the participants had periodontitis, 35.0% had gingivitis, and only 8.8% displayed periodontal health. In 96.4% of the individuals with gingivitis, it was generalized. Of the 45 diabetics with periodontitis, 8 (17.8%) were in stage I, 16 (35.6%) were in stage II, 7 (15.6%) were in stage III, and 14 (31.1%) were in stage IV. Regardless of the stage, the majority had generalized periodontitis. With regard to the progression of periodontitis, 10 (22.2%) were classified as grade B, and 35 (77.8%) were classified as grade C.

Table 5 shows the relationship between periodontal disease and the behavioural factors that modify and aggravate it, as well as the relationship between periodontal disease and patients’ knowledge/information about diabetes and the importance of the dentist in diabetes. There was a statistically significant relationship between periodontal condition and age (χ^2^ (2) = 19.7; *p* < 0.001), with the highest prevalence of periodontitis occurring in individuals over 46 years of age (46.7%). Lower schooling was also significantly associated with periodontal disease (χ^2^ (2) = 10.2; *p* = 0.017), with 35.6% of the 45 individuals with periodontitis having completed secondary school. Being an active smoker and drinking alcohol were not associated with periodontal disease, but it was associated with the number of cigarettes smoked per day (χ^2^ (1) = 6.3; *p* = 0.012) and the frequency of alcohol consumption (χ^2^ (2) = 7.9; *p* = 0.025). The patients’ assessment of their oral condition was statistically significantly related to their periodontal condition (χ^2^ (4) = 15.6; *p* = 0.004). Of the 45 individuals with periodontitis, 13.3% rated their oral condition as very poor and 35.6% as poor, while the periodontally healthy individuals rated only 5 as poor (2.9%) or very poor (11.4%).

When asked “Do you talk to your dentist about diabetes?”, 45.7% of individuals without periodontitis and 51.1% with periodontitis said that they did not. In response to the question “Should dentists talk to you about the importance of good oral health because you have diabetes?”, there was a high degree of congruence in the answers, with 82.9% of those without periodontitis and 91.9% of those with periodontitis saying yes. Exactly the same results were found for the question “Should dentists be involved in diagnosing diabetes?”. Regarding the question “If dentists proposed exams, would you be willing to pay?”, 77.1% of patients without periodontitis and 82.2% of patients with periodontitis answered yes. These findings suggest that patients’ recognition of the dentist’s role in diabetes management is independent of their periodontal condition.

Table 6 shows the relationship between participants’ knowledge of oral health complications and their periodontal condition. No statistically significant association was found between the level of knowledge and the presence of periodontitis. However, individuals with periodontitis more frequently selected broader combinations of oral health problems. In particular, the most commonly cited response, “dry mouth, bad breath, tooth decay, taste changes, bleeding gums when brushing, and delayed healing”, was mentioned by 58.5% of participants with periodontitis, compared to 48.6% of those without the condition. Simpler responses or those indicating a single symptom, such as “tooth decay” or “bleeding gums when brushing”, were either evenly distributed or slightly more common among participants without periodontitis.

### 3.5. Relationship Between Periodontal Disease and Periodontal Parameters

A comparison of periodontal health parameters between individuals with and without periodontitis revealed significant differences across all indices (Table 7). The percentage of PI (plaque index) was significantly higher in the periodontitis group (52.75 ± 28.11) compared to the non-periodontitis group (39.39 ± 24.5; *p* = 0.029). Similarly, the percentage of BOP (bleeding on probing) was greater in individuals with periodontitis (38.31 ± 21.20%) than in those without (23.52 ± 12.98; *p* < 0.001). The total parameters PPD (mm) and CAL (mm) (*p* < 0.001) were also significantly elevated in the periodontitis group (PPD: 3.02 ± 0.97 mm; CAL: 3.05 ± 1.53 mm) compared to the non-periodontitis group (PPD: 2.09 ± 0.37 mm; CAL: 1.58 ± 1.37 mm; *p* < 0.001 for both).

## 4. Discussion

The diabetic population has unique and challenging adversities in terms of general health, such as illnesses, poor eating habits, and poor oral health. Therefore, the promotion of oral and nutritional health becomes a necessity in this population. This study aims to analyze the perceptions of type I diabetic patients about the relationship between diabetes mellitus and periodontal disease.

The current study revealed that the majority of participants were aware of the relationship between diabetes mellitus and periodontal disease, mainly about the oral manifestations associated with diabetes.

### 4.1. Sociodemographic Variables

This study was carried out on 80 adult individuals with type I diabetes diagnosed at the Tâmega e Sousa Hospital Center.

Of the 80 patients who took part in the study, 51 (63.7%) were male and 29 (36.3%) female, which is in line with the study by Habashneh et al. [15], which included 405 participants (213 men and 192 women). However, these data do not coincide with the studies of Banyai et al. [2], Meshki et al. [3], and Paurobally et al. [16], in which the majority of participants were female.

In line with the study carried out by Delmés et al. [21] and Shimpi et al. [13], the participants’ ages ranged from 19 to 72, with the majority (36.3%) aged between 26 and 45. The average age of the patients was 35.9 years, a similar result to the study by Banyai et al. [2]. There was a statistically significant relationship between periodontal condition and age, with the highest prevalence of periodontitis occurring in individuals over 46 years of age.

Our results showed that a lower educational level was significantly associated with periodontal disease. Among the 45 individuals with periodontitis, 35.6% had only completed secondary school. Similarly, in the study by Meshki et al. [3], it was observed that increasing the level of schooling significantly improved knowledge, attitude, and practice in relation to oral health.

### 4.2. Smoking and Oral Hygiene Habits

With regard to oral hygiene habits, the vast majority, 93.8%, reported brushing their teeth daily, with 52.5% doing so twice a day and 93.8% using a toothbrush and toothpaste. These results are in line with the study by Banyai et al. [2], in which 71.3% (*n* = 219) of the participants brushed their teeth twice a day or more, while 24.8% (*n* = 76) performed this routine only once a day. However, in the study by Habashneh et al. [15], 56% of the participants brushed their teeth at least once a day, indicating that regular tooth brushing is not a universal practice in the population surveyed.

As for interdental cleaning, in this study, it was not a common practice among the participants, with 77.5% reporting that they did it rarely, 12.5% sometimes, and 10% daily, and when they did it was through the use of dental floss/brushes, data that are in line with the study by Habashneh et al. [15], where only 35.0% of the interviewees used dental floss or brushes. In contrast to the study by Banyai et al. [2], 60.3% (*n* = 185) of the participants reported using dental floss regularly, which was their favorite interdental cleaning aid.

Our results suggest both a lack of knowledge regarding appropriate interproximal cleaning techniques and insufficient practice in maintaining comprehensive oral hygiene. In this sense, and in line with the study by Habashneh et al. [15], it is necessary to encourage the use of appropriate interproximal hygiene aids, along with education on their use, in order to improve periodontal health and prevent gum disease in this high-risk population.

In our study, we assessed the plaque index and obtained an average percentage of 46.91%. However, despite this figure being high, the results did not show a statistically significant difference between individuals who brushed their teeth once a day, twice a day, or three or more times a day, suggesting that the reliability of the participants’ self-reported brushing habits may be questionable.

According to Kwon et al. [7], certain factors, such as smoking, increase the risk of periodontal disease. Similarly, in our study, we found that smokers were more likely to have periodontitis, although the relationship between smoking and periodontitis was not significant. The number of cigarettes smoked per day was also associated with the prevalence of periodontitis.

### 4.3. Perception of Oral Condition and Frequency of Visits to the Dentist

As for going to the dentist, 20% only do so when they feel pain, 32.5% go once a year, 23.8% twice a year, and 23.8% go more than twice a year. These results are consistent with Banyai et al. [2], where the majority (71.3%) of participants visited the dentist annually. In contrast, the study by Habashneh et al. [15] reported that less than half of their participants had seen a dentist in the last year, with only 10% of the participants attending dental appointments regularly.

As for the reasons for not visiting the dentist more often, we found that the majority (45%) do not see the need to do so, 25% do not because dentists cost too much money, and 17.5% gave another unspecified reason. In this regard, the study by Habashneh et al. [15] said that 80% visited the dentist only when there was a need, 10% reported visiting the dentist every 6–12 months, and 28% reported scheduling visits to check periodontal health.

When asked how they rated the condition of their mouth, only 27 of the participants considered their oral condition to be poor or very poor, while 51.2% said it was good, 11.3% said it was very good, and 3% said it was excellent. The patients’ assessment of their oral condition was statistically significantly related to their periodontal condition; of those who rated their oral condition as very poor or poor (*n* = 27), the majority (*n* = 22) suffered from periodontitis. These results are corroborated by the study by Habashneh et al. [15], where the majority (around 60%) of those interviewed rated their oral health as poor.

### 4.4. Periodontal Diseases

As evidenced in the literature, periodontal health tends to worsen with advancing age and a longer duration of diabetes mellitus. The formation of bacterial plaque and/or tartar can be attributed to various factors, such as negligence in oral hygiene, inadequate hygiene techniques, and limited access to regular dental care [22].

With regard to periodontal diseases, periodontitis was found to be the most prevalent periodontal disease in our sample, with 56.3% of the participants having periodontitis, 35.0% having gingivitis, and only 8.8% displaying periodontal health. According to Banyai et al. [2], Meshki et al. [3], and Delmés et al. [21], several studies have provided evidence that periodontitis is strongly associated with diabetes and oral hygiene.

Out of the 45 diabetics with periodontitis, the majority had generalized periodontitis. With regard to the severity of periodontitis, the most prevalent were stages II and III. Regarding the progression of periodontitis, the majority were classified as grade C.

According to our results, the majority of diabetic patients with periodontitis consider their oral cavity as “good” (46.7%) and do not talk to their dentist about their systemic condition (51.1%). Although the majority of these individuals consider good oral hygiene to be important (91.2%) given their systemic condition and although they consider the involvement of dentists in the diagnosis of periodontal disease to be important (91.2%), there still seems to be a long way to go.

Nevertheless, the diabetic patients with periodontitis who considered that they had a good oral cavity had higher levels of PI compared to the control group (52.75 ± 28.11 vs. 39.39 ± 24.55, respectively). The involvement of microbial plaque in the etiology of PD is unquestionable. Therefore, these individuals are predisposed to greater periodontal inflammation, as evidenced by the increased BOP values seen in these patients, compared to patients who did not have periodontitis (38.31 ± 21.20 vs. 23.52 ± 12.98, respectively). Thus, these individuals are predisposed to a greater degree of periodontal destruction, as evidenced by the increase in BOP, PPD, and CAL which can lead to tooth loss. Similarly, the study by Rigo et al. [23] showed a statistically significant association between those with diabetes and higher levels of probing depth.

Most of the diabetic patients included in this study had periodontitis (56.2%); however, they were not aware of their oral condition, despite the fact that they considered it important to be monitored by a dentist because they had this systemic disease. We can, therefore, conclude that oral health education and the diagnosis and treatment of periodontitis should be recommended as early as possible by health professionals and that communication between the endocrinologist and the dentist should be even closer.

### 4.5. Knowledge of Diabetes-Related Problems and the Importance Assigned to the Dentist

When asked about their knowledge of diabetes-related problems, all the participants showed that they were aware of at least one of these problems, with the majority being aware of 4 or more problems. Similarly, in the study by Paurobally et al. [16], the majority of participants were aware of the association between diabetes and other diseases; however, knowledge of the oral complications of diabetes was limited (caries (29%), periodontal disease (37%), and xerostomia (52%)).

In this study, there was a high level of agreement in the answers to the questions “Should dentists talk about the importance of good oral health because you have diabetes?” and “Should dentists be involved in the diagnosis of diabetes?”, with 82.9% of individuals without periodontitis and 91.9% of those with periodontitis saying yes to both questions, i.e., the importance attributed to the dentist in diabetes is independent of the periodontal condition.

Habashneh et al. [15] argue that dentists have both the responsibility and the opportunity to educate diabetic patients about the oral complications associated with diabetes and to promote appropriate oral health behaviors.

In contrast, the study by Siddiqi et al. [11] reveals that the majority of patients (66.38%) have never discussed their oral health and oral hygiene habits with their endocrinologist, and 54% are unaware of the bidirectional relationship between periodontal disease and diabetes. Only 36.5% reported having received this type of information from their endocrinologist.

According to Paurobally et al. [16], the results seem to indicate that the majority of diabetic patients did not receive guidance/information on oral health from endocrinologists or dentists at the start of their treatment and were not properly informed of their increased risk of complications.

The results showed that there were no statistically significant differences in the knowledge of oral complications of diabetic patients according to periodontal condition.

This assessment helps to identify knowledge gaps and design future interventions to strengthen patients’ understanding of how type I diabetes mellitus and periodontitis are associated. By increasing this knowledge, prevention and appropriate disease management are promoted, ultimately improving patients’ health and quality of life.

### 4.6. Limitations of the Study and Recommendations for Future Research

One limitation of this study was the difficulty in obtaining a representative sample, as data were collected from only one hospital. Additionally, while the use of a questionnaire was cost-effective and practical, the administration of the questionnaire by the researcher may have introduced response bias, as participants could have been reluctant to provide truthful answers due to concerns about judgment.

Another limitation is the lack of formal psychometric validation of the questionnaire. Specifically, internal consistency (e.g., through Cronbach’s alpha) and test–retest reliability were not assessed. Although a pre-test was conducted to evaluate the clarity and comprehension of the questions, future studies should include these measures to strengthen the reliability and reproducibility of the instrument.

It is recommended that future research be carried out on a wider sample of participants, including at several hospital centers in the country.

This study provides evidence of the importance of general oral health awareness and education for diabetic patients. Interdisciplinary cooperation between dentists and other health professionals, particularly endocrinologists, is crucial.

To improve the quality of life of diabetic patients, annual clinical oral examinations should be recommended in their treatment guidelines.

## 5. Conclusions

Type I diabetic patients showed knowledge of the relationship between periodontal diseases and diabetes, and most were aware of the problems related to diabetes, namely oral manifestations.

In general, although many recognize the relationship between diabetes and dental problems, most do not discuss this with their dentist. However, diabetic patients recognize the importance of the dentist providing information about this relationship.

The prevalence of periodontal disease was 56.3%. In terms of severity, the most prevalent was moderate and severe (stage II and III) and rapid progression.

Certain factors, such as lower education, age over 45, and smoking more than 10 cigarettes a day, were positively associated with periodontitis.

The findings emphasize the need for interdisciplinary collaboration between dentists and endocrinologists; patients should be educated about the association between these two chronic inflammatory diseases.

## Figures and Tables

**Table 1:** Demographic characteristics of the study’s participants.

	*n* (%)
Age (mean ± SD)	35.9 ± 13.8
Age, *n* (%)	
≤25 years	27 (33.8)
≥26 years and ≤45 years	29 (36.2)
≥46 years	24 (30.0)
Gender, *n* (%)	
Female	29 (36.3)
Male	51 (63.7)
Education Level, *n* (%)	
Second cycle ^1^	19 (23.8)
Third cycle ^2^	12 (15.0)
Secondary ^3^	27 (33.8)
College ^4^	22 (27.4)
Ocupation, *n* (%)	
Retired	5 (6.2)
Unemployed	3 (3.8)
Student	12 (15.0)
Worker	60 (75.0)
Alcohol Consumption, *n* (%)	
Yes	46 (57.5)
No	34 (42.5)
Frequency of Alcohol Consumption, *n* (%)	
Occasionally	28 (60.9)
1x/day	5 (10.9)
>1x/day	13 (28.3)
Smoking Habits, *n* (%)	
Smoker	17 (21.2)
Non-smoker	63 (78.8)
N° of cigarettes/day, *n* (%)	
≤10 cigarettes	11 (64.7)
>10 cigarettes	6 (35.3)

*n*—number of individuals in the sample; %—percentage; SD—standard deviation; ^1^ 6 years of education; ^2^ 9 years of education; ^3^ 12 years of education; ^4^ higher education.

**Table 2:** Participants’ oral hygiene habits and perception of oral condition, number of dental appointments, and reasons for not going to the dentist.

	*n* (%)
Daily toothbrushing, *n* (%)	
Yes	75 (93.7)
No	5 (6.3)
Frequency of toothbrushing, *n* (%)	
1x day	20 (25.0)
2x day	42 (52.5)
≥3x day	18 (22.5)
Brushing tools, *n* (%)	
Toothbrush	2 (2.4)
Toothpaste	3 (3.8)
Toothbrush + paste	75 (93.8)
Dental floss/interdental brushes, *n* (%)	
Yes	22 (27.5)
No	58 (72.5)
Interdental cleaning frequency, *n* (%)	
Rarely	62 (77.5)
Sometimes	10 (12.5)
Daily	8 (10.0)
Tongue brushing, *n* (%)	
Yes	41 (51.3)
No	23 (28.7)
Sometimes	16 (20.0)
Use of dentures, *n* (%)	
Yes	12 (15.0)
No	68 (85.0)
Perception of oral condition, *n* (%)	
Very weak and weak	27 (33.8)
Good	41 (51.2)
Very good	9 (11.3)
Excellent	3 (3.7)
Visits to the dentist/hygienist, *n* (%)	
When pain occurs	16 (20.0)
1x year	26 (32.4)
2x year	19 (23.8)
>2x year	19 (23.8)
Reasons for not going to the dentist, *n* (%)	
Do not see the need for it	36 (45.1)
Spend a lot of money	20 (25.0)
Another reason	14 (17.5)
Fear or anxiety	3 (3.8)
Do not like dentists	3 (3.8)
Going to dentist is unpleasant	1 (1.2)
Fear or needing treatment	1 (1.2)
Do not remember going to the dentist	1 (1.2)
Difficulty of transport	1 (1.2)
Heathcare system, *n* (%)	
Private clinics	59 (73.8)
National Health System	18 (22.5)
Mixed system	3 (3.7)

*n* = frequency; % = percentage.

**Table 3:** Responses of the participants regarding oral health and its importance in diabetes.

Knowledge of Diabetes-Related Problems, *n* (%)	*n* (%)
Tooth decay	4 (5.0)
Bleeding gums when brushing + tooth decay + bad breath	5 (6.3)
Bleeding gums when brushing	5 (6.3)
Dry mouth + tooth decay + gums bleed when brushed + swollen or sensitive gums	6 (7.5)
Healing difficulties in the mouth + gums bleed when brushed + swollen or sensitive gums + difficulty in healing the mouth	8 (10.0)
Dry mouth	11 (13.8)
Dry mouth + bad breath + tooth decay + taste changes + bleeding gums when brushing + difficulty in healing the mouth	41 (51.1)
Have you been told by a health professional to take the following precautions, *n* (%)	*n* (%)
Flossing	2 (2.5)
Brushing teeth	2 (2.5)
Regular visits to the dentist + flossing	1 (1.2)
Brushing teeth + flossing	1 (1.2)
Brushing teeth + controlling the gums	1 (1.2)
Brushing teeth + visiting the dentist regularly	11 (13.8)
Brushing teeth + visiting the dentist regularly + controlling the gums	15 (18.8)
Brushing teeth + visiting the dentist regularly + flossing	6 (7.5)
Brushing teeth + controlling the gums + flossing	1 (1.2)
Brushing teeth + visiting the dentist regularly + controlling the gums + visiting the hygienist regularly + flossing	25 (31.3)
None of the above	15 (18.8)
Do you discuss diabetes with your dentist?, *n* (%)	*n* (%)
No	39 (48.8)
Sometimes	22 (27.5)
Frequently	19 (23.7)
The importance of the dentist in addressing oral health, *n* (%)	
No	6 (7.5)
I do not know	4 (5.0)
Yes	70 (87.5)
Should dentists be involved in diagnosing diabetes?, *n* (%)	*n* (%)
No	6 (7.5)
I do not know	4 (5.0)
Yes	70 (87.5)
If the dentist proposed exams requiring payment, would you be willing to pay?, *n* (%)	*n* (%)
No	6 (7.5)
I do not know	10 (12.5)
Yes	64 (80.0)

*n* = frequency; % = percentage.

**Table 4:** Periodontal status of type I diabetic patients.

	*n* (%)
Periodontal health	7 (8.8)
Gengivitis	28 (35.0)
Localized	1 (3.6)
Generalized	27 (96.4)
Periodontitis	45 (56.2)
Stage I	8 (17.8)
Generalized	5 (62.5)
Localized	3 (37.5)
Stage II	16 (35.5)
Generalized	13 (81.3)
Localized	3 (18.7)
Stage III	7 (15.6)
Generalized	7 (100.0)
Localized	0 (0.0)
Stage IV	14 (31.1)
Generalized	14 (100.0)
Localized	0 (0.0)
Grade B	10 (22.2)
Grade C	35 (77.8)

*n* = frequency; % = percentage.

**Table 5:** Relationship between periodontal disease and the behavioral factors that modify and aggravate it, and the importance of the dentist in diabetes.

	Without Periodontitis (*n* = 45)	With Periodontitis (*n* = 35)		
	*n* (%)	*n* (%)	χ^2^	*p*
Gender				
Female	16 (45.7)	13 (28.9)	2.4	0.120
Male	19 (54.3)	32 (71.1)		
Age				
≤25 years	20 (57.1)	7 (15.6)	19.7	<0.001
≥26 ≤45 years	12 (34.3)	17 (37.7)		
≥46 years	3 (8.6)	21 (46.7)		
Educational level				
Second cycle ^1^	3 (8.6)	16 (35.6)	10.2	0.017
Third cycle ^2^	4 (11.4)	8 (17.8)		
Secondary ^3^	15 (42.9)	12 (26.6)		
College ^4^	13 (37.1)	9 (20.0)		
Active smoker				
Yes	5 (14.3)	12 (26.7)	1.8	0.179
No	30 (85.7)	33 (73.3)		
Number of cigarettes/day				
≤10 cigarettes/day	0 (0.0)	8 (66.7)	6.3	0.012
>10 cigarettes/day	5 (100.0)	4 (33.3)		
Alcohol consumption				
Yes	18 (51.4)	28 (62.2)	0.9	0.333
No	17 (48.6)	17 (37.8)		
Frequency of alcohol consumption				
Ocasionaly	14 (77.8)	14 (50.0)	7.9	0.025
1x week	3 (16.7)	2 (7.1)		
≥1x day	1 (5.5)	12 (42.9)		
Oral hygiene habits				
1x day	7 (20.0)	13 (27.7)	0.95	0.623
2x day	19 (54.3)	23 (52.3)		
≥3x day	9 (25.7)	9 (20.0)		
How do you evaluate your oral condition?				
Very weak	1 (2.9)	6 (13.3)	15.6	0.004
Weak	4 (11.4)	16 (35.6)		
Good	20 (57.1)	21 (46.7)		
Very good	8 (22.9)	1 (2.2)		
Excellent	2 (5.7)	1 (2.2)		
Do you speak to your dentist about diabetes?				
No	16 (45.7)	23 (51.1)	3.4	0.186
Sometimes	13 (37.2)	9 (20.0)		
Frequently	6 (17.1)	13 (28.9)		
Should dentists talk about the importance of good oral health, because you have diabetes?				
No	4 (11.4)	2 (4.4)	1.5	0.473
Yes	29 (82.9)	41 (91.2)		
I do not know	2 (5.7)	2 (4.4)		
Should dentists be involved in diagnosing diabetes?				
No	4 (11.4)	2 (4.4)	1.5	0.473
Yes	29 (82.9)	41 (91.2)		
I do not know	2 (5.7)	2 (4.4)		
If the dentists suggest exams, would you be willing to pay?				
No	3 (8.6)	3 (6.7)	0.32	0.853
Yes	27 (77.1)	37 (82.2)		
I do not know	5 (14.3)	5 (11.1)		

*n* = frequency; % = percentage; *p* = significance level; χ^2^ = chi-square; ^1^ 6 years of education; ^2^ 9 years of education; ^3^ 12 years of education; ^4^ higher education.

**Table 6:** Relationship between knowledge of diabetes-related problems and periodontal disease.

Knowledge of Diabetes-Related Problems	Without Periodontitis (*n* = 45)	With Periodontitis (*n* = 35)		
	*n* (%)	*n* (%)	χ^2^	*p*
Dental caries	2 (5.7)	2 (4.4)	1.69	0.946
Dry mouth	4 (11.4)	7 (15.6)		
Bleeding gums when brushing	3 (8.6)	2 (4.4)		
Bleeding gums + dental caries + bad breath	3 (8.6)	2 (4.4)		
Delayed healing + bleeding gums + bad breath + dry mouth	3 (8.6)	5 (11.1)		
Dry mouth + bad breath + dental caries + swollen/sensitive gums	3 (6.6)	3 (6.7)		
Dry mouth + bad breath + dental caries + taste changes + bleeding + delayed healing	17 (48.6)	24 (58.5)		

**Table 7:** Periodontal indices in individuals with and without periodontitis.

	With Periodontitis (*n* = 45)	Without Periodontitis (*n* = 35)			
	Mean ± SD	Mean ± SD	*t*	*p*	Cohen’s d
PI	52.75 ± 28.11	39.39 ± 24.55	2.23	0.029	0.94
BOP	38.31 ± 21.20	23.52 ± 12.98	3.63	<0.001	0.92
PPD Total mm	3.02 ± 0.97	2.09 ± 0.37	5.38	<0.001	1.6
CAL Total mm	3.05 ± 1.53	1.58 ± 1.37	4.44	<0.001	1.5

Mean ± SD = mean (standard deviation); *t* = *t*-statistic derived from the independent *t*-test *p* = significance level; d = effect size; PI: plaque index; BOP: bleeding on probing; PPD: periodontal probing depth; CAL: clinical attachment loss.

## Data Availability

The data can be accessed by contacting the corresponding author.

## Abbreviations

The following abbreviations are used in this manuscript:

PPD	Periodontal probing depth
REC	Gingival recession
CEJ	Cementoenamel junction
CAL	Clinical attachment loss
PI	Plaque index
BOP	Bleeding on probing
AAP	American Association of Periodontology
EFP	European Federation of Periodontology
*n*	Number of individuals in the sample
%	Percentage
SD	Standard deviation
*p*	Significance level
X^2^	Chi-square
*t*	Statistic derived from the independent t-test
d	Effect size
